# *SPTAN1*, *APC*, *and FGFR3* Mutation Status and APOBEC Mutation Signatures are Predictive of Mitomycin C Response in Non-muscle-invasive Bladder Cancer

**DOI:** 10.1016/j.euros.2021.09.018

**Published:** 2021-11-05

**Authors:** Maria Skydt Lindgren, Philippe Lamy, Sia Viborg Lindskrog, Emil Christensen, Iver Nordentoft, Karin Birkenkamp-Demtröder, Benedicte Parm Ulhøi, Jørgen Bjerggaard Jensen, Lars Dyrskjøt

**Affiliations:** a Department of Urology, Aarhus University Hospital, Aarhus, Denmark; b Department of Urology, Gødstrup Hospital, Gødstrup, Denmark; c Department of Molecular Medicine, Aarhus University Hospital, Aarhus, Denmark; d Department of Clinical Medicine, Health, Aarhus University, Aarhus, Denmark; e Department of Pathology, Aarhus University Hospital, Denmark

**Keywords:** Biomarkers, Bladder cancer, Chemoablation, Chemoresection, Intravesical instillations, Mitomycin C, Non–muscle-invasive bladder cancer, Predictive biomarkers, Urothelial carcinoma

## Abstract

**Background:**

Currently, no biomarkers of response to mitomycin C have been identified in non–muscle-invasive bladder cancer patients. Predictive biomarkers could improve the treatment outcome and eliminate adverse events from unnecessary treatment.

**Objective:**

To identify and validate predictive biomarkers of chemoresection with mitomycin C.

**Design, setting, and participants:**

The intervention group of a randomised controlled trial was identified for analyses. The study was conducted between January 2018 and June 2019 in two major urological departments in Denmark. Patients had a history of Ta low-grade/high-grade disease and were included upon recurrence. The intervention group (58 patients) received chemoresection with mitomycin C. Tumour and reference germline DNA from prior tumours were analysed by whole exome sequencing. Predictive biomarkers were validated in the context of Ta low-grade tumours from the UROMOL study.

**Outcome measurements and statistical analysis:**

Response to chemotherapy (intervention group from the randomised controlled trial) and recurrence-free survival (UROMOL cohort) were measured. Groups were compared using Fisher’s exact test and Wilcoxon rank sum test.

**Results and limitations:**

Chemoresponse was associated with the mutation status of *SPTAN1*, *APC*, and *FGFR3*, and the level of APOBEC signature contribution (*p* = 0.035, *p* = 0.034, *p* = 0.055, and *p* = 0.035, respectively). The main limitations include no biopsy for biomarker discovery immediately prior to chemoresection and the unmatched validation cohort.

**Conclusions:**

Mutation status of *APC, SPTAN1*, *and FGFR3* and the level of mutational contribution from APOBEC-related signatures were identified as potential predictive biomarkers for chemoresection with mitomycin C in non–muscle-invasive bladder cancer patients. A prospective validation study is however needed.

**Patient summary:**

We investigated DNA from noninvasive bladder tumours in order to predict treatment response to chemotherapy. Four biomarkers showed promising results, which should be tested in future studies.

## Take Home Message

Four promising biomarkers were found to be predictive for intravesical mitomycin C in non–muscle-invasive bladder cancer patients. Prospective validation is needed.

## 1 Introduction

Non–muscle-invasive bladder cancer (NMIBC) is a heterogeneous disease with highly disparate prognoses [1], [2]. Hence, generalised adjuvant treatment of this patient group will inevitably result in overtreatment in some while ineffective in others. Currently, we are unable to identify patients who will benefit from mitomycin C (MMC). The discovery of several distinct molecular signatures and subtypes in NMIBC provides a more accurate disease prognosis in addition to clinicopathological features [3]. Molecular characterisations could potentially explain why clinicopathologically similar tumours can display very different disease courses. However, so far, none of the molecular subtypes described in NMIBC have shown a predictive value. Optimally, we need predictive biomarkers in NMIBC to stratify individual patients to the appropriate adjuvant treatment: surveillance alone, MMC, or bacillus Calmette-Guérin (BCG).

To comply with the elderly and often multimorbid patients with NMIBC, we have investigated previously whether patients with intermediate- and high-risk Ta tumours could be spared from surgery [4]. We studied the effect of monotherapeutic chemoresection with MMC compared with surgery and adjuvant instillations. Chemoresection with MMC was found to be effective in 57% of patients and showed fewer side effects. Ideally, this treatment could be offered to patients with chemosensitive tumours, while surgery as monotherapy or in combination with BCG could be offered to patients with chemoresistant tumours. However, the molecular mechanisms of chemoresistance are currently poorly understood, and no research exists on predictive biomarkers in NMIBC patients treated with MMC [5].

The aim of the current study was to assess predictive tumour biomarkers of chemoresection with MMC in patients with recurrent NMIBC.

## 2 Patients and methods

The study population consisted of the intervention group of a randomised, controlled trial conducted between January 2018 and June 2019 [4]. The intervention group consisted of 58 chemo-naïve patients with a known history of Ta bladder tumours (low or high grade), who were included upon recurrence with multiple tumours smaller than 2 cm. All patients received short-term intensive chemoresection with MMC: three weekly instillations with MMC (40 mg/40 ml) for 2 wk. Treatment response was evaluated by flexible cystoscopy 4 wk after treatment completion by visual assessment. The study was approved by the regional ethics committee (1-10-72-148-17) and the Danish Medical Authorities (2017054019), registered with clinicaltrials.gov (NCT03348969), and monitored by Good Clinical Practice. REMARK guidelines were followed for reporting [6].

### 2.1 Biological material

The latest resected tumour prior to study inclusion was collected from all patients (chemo-naïve samples). Moreover, residual tumour tissue after chemoresection was collected from nonresponding patients (postchemo samples). All specimens were formalin fixed and paraffin embedded (FFPE). Carcinoma cells were marked on haematoxylin and eosin–stained overview sections. Germline DNA was extracted from peripheral blood leucocytes or from smooth muscle cells in FFPE blocks using the QIAsymphony DSP DNA midi kit (cat#937255; Qiagen, Hilden, Germany).

### 2.2 Exome sequencing

A detailed description is published elsewhere [7]. In brief, the Twist Human Core Exome EF Multiplex Complete Kit (PN 100795; Twist Bioscience, San Francisco, CA, USA) was used for whole exome sequencing (WES). Input for libraries was 50 ng DNA from tumour and blood. Libraries were sequenced on the Illumina Novaseq 6000 platform (2 × 150 bp paired end; Illumina Inc., San Diego, CA, USA). All somatic alterations were annotated using SnpEff and hg38 build. Additionally, for DNA damage response (DDR) genes, PolyPhen-218 and MutationAssessor were used to classify alterations as damaging or not. In our statistical analyses, we focused on genes previously reported to be associated with bladder cancer by comparison with The Cancer Genome Atlas Bladder Cancer project, the Memorial Sloan Kettering Cancer Center cBioPortal for Cancer Genomics, and other previously published data [3], [8].

### 2.3 Validation cohort

No research on predictive biomarkers of MMC in NMIBC exists; thus, finding a similar validation cohort was impossible. Instead, we identified Danish patients with Ta low-grade disease in the UROMOL cohort for validation of the mutation status of candidate genes (*SPTAN1*, *APC*, *FGFR3*, *PARP4*, *AFDN*, and *ZFHX3)* and their correlation with recurrence-free survival (RFS) [3]. *PTEN* was not included in the validation as *PTEN* mutations were called only in two patients in the validation cohort. In this cohort, 25% of the patients were confirmed to have received MMC in relation to the analysed tumour, and RFS was used as a proxy for tumour response. Differences between patients who have received MMC and those who have not could be a measure for predictive biomarkers as opposed to prognostic markers. Mutation calls derived from RNA sequencing (*n* = 209). DNA hotspot mutation analysis was available for *FGFR3* only (*n* = 162). In this group, 28% of patients were confirmed to have received MMC in relation to the analysed tumour. Data from the validation cohort are approved for analyses by the Danish National Committee on Health Research Ethics (#1906019).

### 2.4 Statistical analyses

Genes associated with treatment response were analysed using Fisher’s exact test. The mutational contribution and the mutational counts in a signature context were compared between groups by the Wilcoxon rank sum test. RFS was analysed using the Kaplan-Meier method and tested by the log-rank test. Patients lost to follow-up were censored. No correction for multiple testing was applied due to confinement of the analyses to candidate genes as mentioned above and due to the low sample size.

## 3 Results

**Fig. 1:**
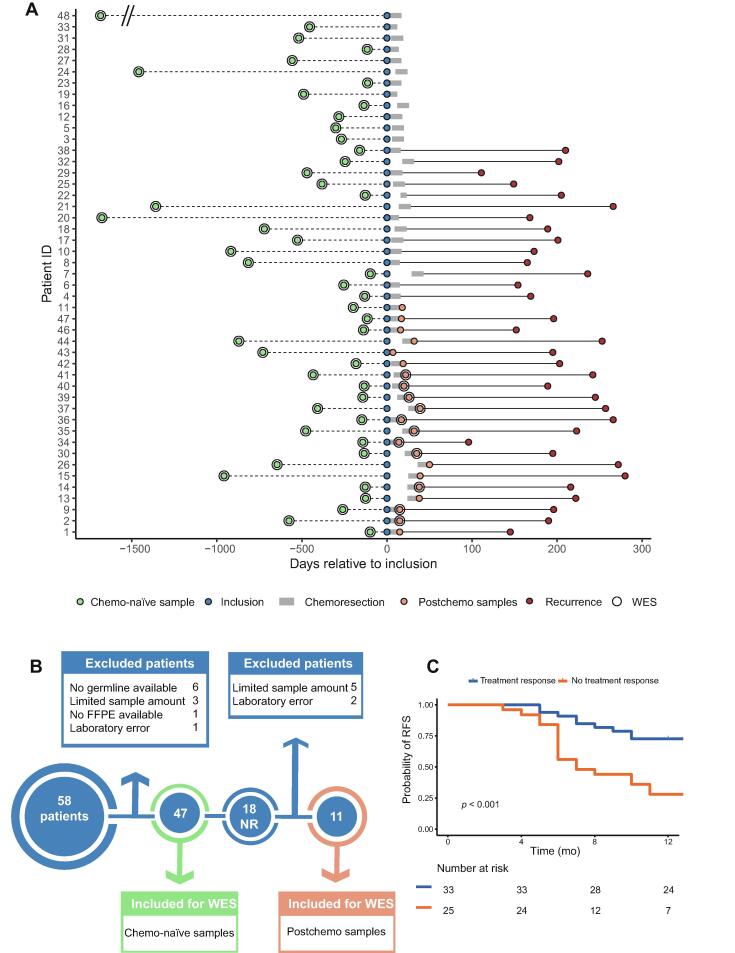
Clinical characteristics of analysed patients. (A) Tumour timeline. Chemo-naïve samples are tumours removed prior to study inclusion. For illustrative purposes, the dashed line is divided by two for ID 48. (B) Flowchart for exome sequencing analysis. (C) Recurrence-free survival (RFS) estimates by treatment response to chemoresection. FFPE = formalin-fixed paraffin-embedded block; NR = nonresponding patients; RFS = recurrence-free survival; WES = whole exome sequencing.

In total, 47 patients contributed with 47 chemo-naïve tumour samples and 11 postchemo tumour samples (Fig. 1A). See Figure 1B for a tumour flowchart. Patients with a complete response to chemoresection had significantly longer RFS than patients with no response (Fig. 1C). Tumour samples were sequenced to a mean coverage of 121X (range: 74–212) for chemo-naïve samples, 131X (range: 50–220) for postchemo samples, and 130X (range: 20–220) for reference germline samples. In chemo-naïve samples, a median of 172 single-nucleotide variants (SNVs; range: 14–962) and 55 insertions or deletions (indels; range: 3–916) were identified per patient. In postchemo samples, a median of 149 SNVs (range: 26–772) and 44 indels (range: 5–425) were identified per patient.

### 3.1 Biomarker discovery

**Fig. 2:**
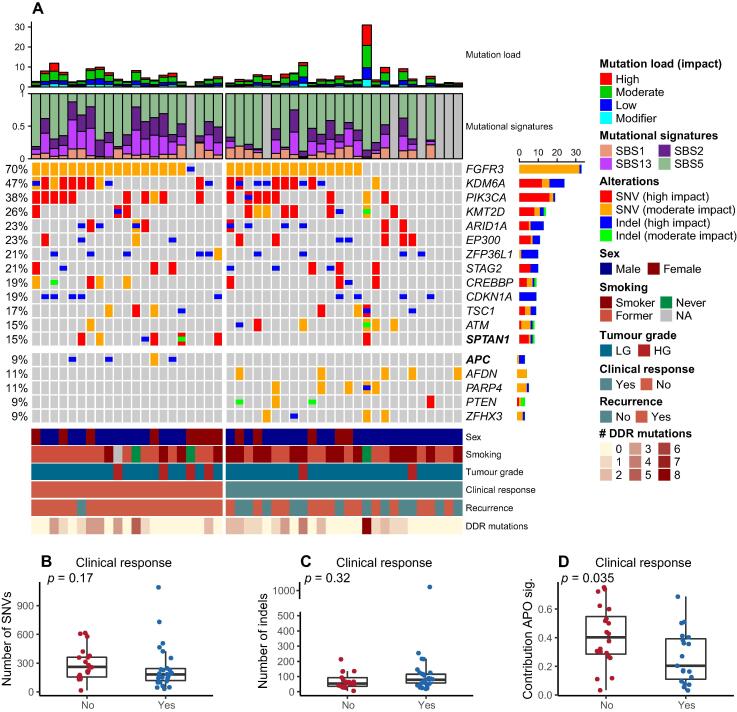
Clinical characteristics and genomic alterations associated with response to chemoresection. (A) Oncoplot showing the most frequently mutated genes, known to be associated with bladder cancer in the 47 chemo-naïve tumour samples. Genes listed in bold have a statistically significant association with treatment response. The top panels are annotated by mutation load, which is impact stratified according to SnpEff and mutational signatures. The bottom panel is annotated by clinical and histopathological characteristics, treatment response, recurrence during 12 mo of follow-up, and the number of DDR-related genes with protein damaging mutations. (B and C) Boxplots showing the total number of SNVs and indels by treatment response. (D) Boxplot showing the APOBEC signature contribution by treatment response. DDR = DNA damage response; indel = insertion or deletion; NA = not applicable; SBS = single-base substitution; SNV = single-nucleotide variant.

Figure 2A outlines the most frequently mutated genes and genes where the mutation frequency differs between response groups, together with clinical and histopathological characteristics. The most frequently mutated genes in the chemo-naïve samples were well-established bladder cancer–associated genes: *FGFR3* (70%), *KMD6A* (47%), *PIK3CA* (38%), *KMT2D* (26%), and *ARID1A* (23%). Overall, we observed no significant difference between chemosensitive and chemoresistant tumours regarding the number of SNVs or indels (Fig. 2B and C).

Chemo-naïve samples were then analysed to identify predictive biomarkers of chemoresection with MMC. When comparing the differences in gene mutation frequencies between responding and nonresponding patients, we found seven genes to be of interest: mutations in *SPTAN1*, a gene involved in DDR, and the tumour suppressor gene *APC* were significantly associated with chemoresistant tumours (*p* = 0.035 and 0.034, respectively). Wild-type *FGFR3* was associated with chemosensitive tumours (*p* = 0.055). Furthermore, mutations in *PARP4*, *AFDN*, *ZFHX3*, and *PTEN* occurred in responding patients only (*p* = 0.056, *p* = 0.056, *p* = 0.1, and *p* = 0.1, respectively; Fig. 2A). The mutational landscape was further investigated by analysing previously identified bladder cancer–associated mutational signatures: single-base substitution 1 (SBS1), SBS2, SBS5, and SBS13 [9]. SBS2 and SBS13 are associated with APOBEC mutagenesis and have previously been linked to carcinogenesis in bladder cancer [10]. The mutational contribution from APOBEC signatures (SBS2 + SBS13) was significantly higher in nonresponding patients than in responding patients (*p* = 0.035; Fig. 2D). We also observed a higher absolute number of mutations in an APOBEC signature context in chemoresistant tumours than in chemosensitive tumours, but it reached statistical significance (*p* = 0.09). SBS5 is a signature related to *ERCC2* mutations and proposedly other DDR pathways [11]. There was no difference in the absolute number of mutations contributed from SBS5 between responding and nonresponding patients (*p* = 0.4). In continuation, there was no association between protein damaging mutations in DDR-related genes and response to chemoresection (Fig. 2A).

### 3.2 Independent validation

**Fig. 3:**
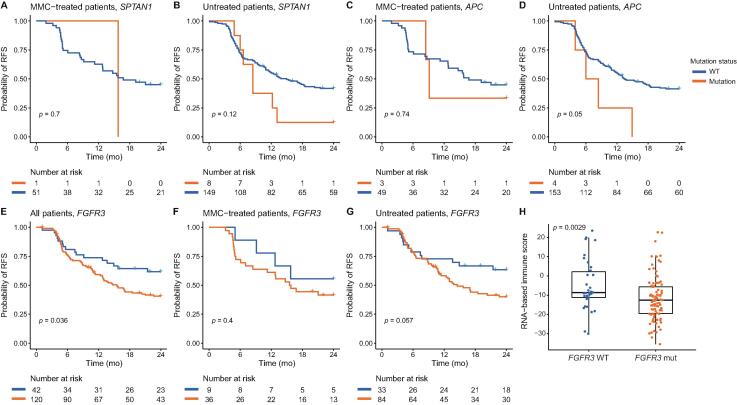
Independent validation: (A–G) Kaplan-Meier plots showing the recurrence-free survival estimates in the UROMOL cohort by gene mutation status for patients treated both with and without MMC. (H) RNA-based immune score by FGFR3 mutation status in the UROMOL cohort. MMC = mitomycin C; RFS = recurrence-free survival; WT = wild type.

No associations between 24-mo RFS and the mutation status of the candidate genes were found (Fig. 3A–D, results shown for *SPTAN1* and *APC*). *FGFR3* mutation status was significantly associated with the 24-mo RFS in all patients (*p* = 0.036), and not in the MMC-treated patients alone (*p* = 0.4; Fig. 3E–G).

As the effect of chemotherapy has been linked to the patient's immune system, we investigated the correlation with an immune infiltration score that estimates the presence of immune cells in the tumour microenvironment [3], [12], [13]. Importantly, we observed that wild-type *FGFR3* tumours in the UROMOL validation cohort were significantly associated with a higher RNA-derived immune infiltration score (*n* = 129; Wilcoxon rank sum test, *p* = 0.003; Fig. 3H), indicating a tumour microenvironment with an immune system supportive of response. Trinucleotide signatures in the UROMOL cohort were called based on RNA-sequencing data, for which the methodology was different from that for DNA-based signature calls. The resulting APOBEC-like signatures from the RNA-sequencing data showed no association with RFS in the UROMOL cohort.

### 3.3 Clonal evolution following MMC treatment

Eleven nonresponding patients had paired chemo-naïve and postchemo samples (Supplementary Fig. 1). Overall, we observed no difference in the median number of SNVs or indels between chemo-naïve and postchemo samples.

**Fig. 4:**
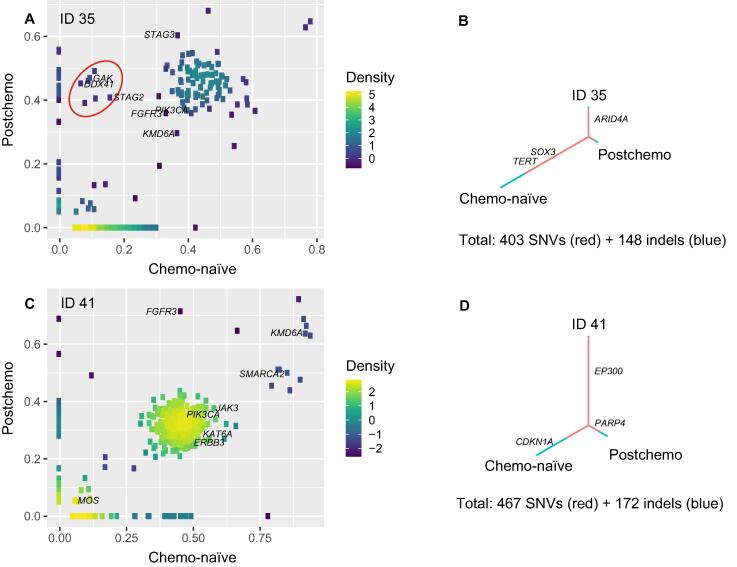
Clonal evolution following MMC treatment. (A and C) Scatter plots of allele frequencies of mutations in chemo-naïve and postchemo samples for ID 35 and ID 41. (B and D) Tumour evolution trees inferred by SNVs and indels for ID 35 and ID 41. The vertical line shows the proportion of shared mutations between chemo-naïve and postchemo samples. The proportions of private mutations are shown in the branches. The total number of mutations is listed at the bottom of each tree. Indel = insertion or deletion; MMC = mitomycin C; SNV = single-nucleotide variant.

Subsequently, we investigated whether subclonal tumour cell populations sensitive to MMC would disappear during chemoresection, while subclonal tumour cell populations resistant to MMC would persist. All mutations were therefore categorised as shared mutations or mutations private to either chemo-naïve or postchemo samples. ID 35 experienced a tumour burden progression during chemoresection; we observed a change in the composition of mutations shared between samples before and after chemoresection. The change in allele frequency observed in a selected group of mutations (Fig. 4A; red circle) demonstrates how these mutations constitute only a small fraction of mutations in the chemo-naïve sample (low allele frequency) but a large fraction in the postchemo sample (high allele frequency), indicating that a subclone had become dominant. In addition, the postchemo sample was less heterogeneous than the chemo-naïve sample, indicated by the small proportion of private postchemo mutations in Figure 4B. Hence, fewer mutations were present after chemoresection. Different results are observed for ID 41, for whom the postchemo tumour showed no visual change after chemoresection. This is illustrated in Figure 4C and D, where no substantial changes are seen. Here, the allele frequencies are similar, and a large fraction of mutations are shared between chemo-naïve and postchemo samples, although the postchemo sample is somewhat less heterogeneous. These patterns were, however, not persistent in all patients.

## 4 Discussion

Bladder cancer is a heterogeneous disease, and large patient cohorts are required for identifying robust biomarkers [14]. Here, we set out to delineate biomarkers based on a relatively small, but clinically well-defined cohort. The strength and novelty of this study are the direct response measures, which allowed us to investigate true predictive biomarkers.

In the present study, we observed an association between MMC treatment response and the mutation status of *SPTAN1*, *APC*, *FGFR3*, and APOBEC-related signatures. The results on *FGFR3* did not meet statistical significance (*p* = 0.055), but independent validation showed consistent results for patients treated both with and without MMC (*p* = 0.036). Biomarkers found in the UROMOL validation cohort could be both predictive and prognostic; we do not know whether patients are recurrence free because of the intravesical MMC or because they harbour tumours of good prognosis. This could explain the lack of consistency with the validation cohort, which however could also be due to type I statistical error, even though only candidate genes were investigated in the discovery cohort. The validation is furthermore challenged by the low number of patients who have received MMC in the validation cohort. Based on the imperfectly matched validation cohort, the results need further validation.

Interestingly, no association was observed between the mutation status of DDR-related genes and treatment response, even though MMC exerts its cytotoxicity through DNA cross-linkage. However, both *SPTAN1* and *APC,* which were significantly associated with response, and *PARP4,* which was mutated in chemosensitive tumours only, have been described as DDR-involved genes [15], [16]. Zhang et al. [15] showed that repressing *SPTAN1* led to increased MMC-induced chromosomal instability and hence treatment response in lung cancer. This supports our results, implying that the mutations found in *SPTAN1* are activating. *APC* mutations have been shown to induce chemoresistance in numerous cancer forms through several mechanisms within the cancer cell. Among other mechanisms, *APC*, in in vivo studies, is shown to regulate the nonhomologous end joining repair system that is involved in the repair of DNA double-strand breakage. Furthermore, *APC* mutations have been shown to induce chemoresistance through angiogenesis by interacting with the tumour microenvironment. This is supportive of a connection between *APC* mutation status and MMC resistance, considering that MMC works through reduction that is inhibited by oxygen and hence blood supply [17], [18]. Lastly, in vivo studies showed that loss of *APC* was associated with chemoresistance through alterations of the immune cell infiltration [17].

A corresponding interaction between the immune system and the response to chemotherapy could be responsible for the MMC sensitivity seen in wild-type *FGFR3* tumours in the present study. In the UROMOL cohort, wild-type *FGFR3* was associated with tumours infiltrated by immune cells, which is in line with other studies [19], [20]. Further support is found in muscle-invasive bladder cancer (MIBC), where immune cell infiltration was found to be associated with a response to chemotherapy [7]. In continuation, a growing body of evidence indicates that the antitumour activities of chemotherapy are reliant on the patient’s immune system [13], [21].

The mechanism of chemoresistance is certainly complex, and additional information could be gained from analysing mutational signatures to the mutation status of candidate genes. The published data on APOBEC-related signatures and treatment response are conflicting [22]. Tumours with APOBEC-induced mutagenesis are suggested to be mutated widely, more heterogeneous, and hence more treatment resistant than tumours with no APOBEC-related mutagenesis [23]. In MIBC, a high APOBEC mutational contribution has been associated with a poor response to neoadjuvant chemotherapy [7]. Furthermore, an in vitro study found increased MMC resistance in cell lines with APOBEC mutagenesis activity [24]. On the contrary, APOBEC-related T1 high-grade tumours have been reported to have a good outcome [25]. Furthermore, APOBEC-related tumours have been shown to have a higher tumour mutation burden [26], which in MIBC has been associated with a good response to immune therapy [27]. In the present study, a high level of ABOBEC-related signatures could be a marker for chemotherapy resistance rather than for MMC resistance specifically.

We found no substantial change in the number of mutations between chemo-naïve and postchemo samples, indicating that MMC treatment did not result in any substantial clonal selection or increased mutagenesis. The proportion of shared mutations between chemo-naïve and postchemo samples, however, ranged widely, which could rely on both sampling issues and tumour evolution prior to or during MMC treatment. We would, however, expect the mutations of chemosensitive cells to be present in chemo-naïve samples only. In continuation, we would expect that tumours developing during chemoresection would be more homogeneous: only harbouring mutations of chemoresistant cells, as depicted in the postchemo sample of ID 35. Furthermore, we would expect the mutations of a tumour unaffected by treatment to be generally unchanged, as seen for ID 41. The small decrease in heterogeneity in the postchemo sample could rely on sampling or that some chemosensitive cells disappeared during treatment after all.

Owing to a high mutation frequency, bladder cancer can exhibit considerable temporal heterogeneity, which imposes a major limitation to the study, as no immediate pretreatment biopsy was taken. For maximum comparability, the latest resected tumour prior to study inclusion was therefore investigated. We chose not to take a pretreatment biopsy to avoid tumour ablation not derived from chemoresection.

Another limitation is the validation of the present study. We were not able to confirm the MMC treatment status for all patients in the UROMOL cohort, and the response measure was RFS. The biomarkers observed could therefore be both predictive and prognostic. However, we hypothesise that RFS is a good proxy for MMC efficacy in this patient cohort. Further validation is however needed from a prospective cohort treated with chemoresection.

## 5 Conclusions

*APC*, *SPTAN1*, and *FGFR3* mutation status and the level of mutational contribution from APOBEC-related signatures were identified as potential predictive biomarkers for chemoresection with MMC in NMIBC patients. A prospective validation study is however needed.

  ***Author contributions:*** Maria Skydt Lindgren had full access to all the data in the study and takes responsibility for the integrity of the data and the accuracy of the data analysis.

  *Study concept and design*: Jensen, Dyrskjøt, Lindgren.

*Acquisition of data*: Lindgren, Birkenkamp-Demtröder, Nordentoft, Lamy.

*Analysis and interpretation of data*: Lindgren, Lamy, Lindskrog, Christensen, Dyrskjøt.

*Drafting of the manuscript*: Lindgren.

*Critical revision of the manuscript for important intellectual content*: Dyrskjøt, Jensen, Lamy, Lindskrog, Nordentoft.

*Statistical analysis*: Lindgren, Lamy, Lindskrog, Christensen.

*Obtaining funding*: Jensen, Lindgren.

*Administrative, technical, or material support*: Birkenkamp-Demtröder, Nordentoft, Lamy, Ulhøi.

*Supervision*: Dyrskjøt, Jensen.

*Other*: None.

  ***Financial disclosures:*** Maria Skydt Lindgren certifies that all conflicts of interest, including specific financial interests and relationships and affiliations relevant to the subject matter or materials discussed in the manuscript (eg, employment/affiliation, grants or funding, consultancies, honoraria, stock ownership or options, expert testimony, royalties, or patents filed, received, or pending), are the following: Jørgen Bjerggaard Jensen: proctor—Intuitive Surgery; member of advisory boards—Ferring, Cepheid, Roche, Ambu, and Olympus; speaker—Medac and Olympus; research collaboration—Photocure ASA, Medac, Roche, Ferring, Karl Storz, Olympus, Intuitive Surgery, Astellas, AstraZeneca, Cephaid, EpiCheck, Pfizer, and Urotech; patent—JB-One en bloc retriever. Lars Dyrskjøt: member of advisory boards—BioXpedia A/S; research collaboration—C2i, AstraZeneca, Natera, and Ferring; advisor/consult—Ferring.

  ***Funding/Support and role of the sponsor:*** The Danish Cancer Society, Medac GmbH Germany, the Foundation of Erik & Susanna Olesen, and the Foundation of Engineer H. C. Bechgaard & Ella Mary Bechgaard supported this study.

## Appendix A Supplementary material

Supplementary Fig. 1 Clinical characteristics and genomic alterations from 11 non-responding patients with paired chemo-naïve and post-chemo samples. A. Oncoplot showing the most frequently mutated genes, known to be bladder cancer-associated in 11 paired chemo-naïve and post-chemo tumour samples. The top panels are annotated by mutation load, which is impact-stratified according to SnpEff and mutational signatures. The bottom panel is annotated by clinical and histopathological characteristics.
