# Supplementary figures and images for: SPTAN1, APC, and FGFR3 Mutation Status and APOBEC Mutation Signatures are Predictive of Mitomycin C Response in Non-muscle-invasive Bladder Cancer
Source: Eur Urol Open Sci. 2021 Nov 5;34:59–67. doi: 10.1016/j.euros.2021.09.018 (PMC8655384; doi:10.1016/j.euros.2021.09.018)

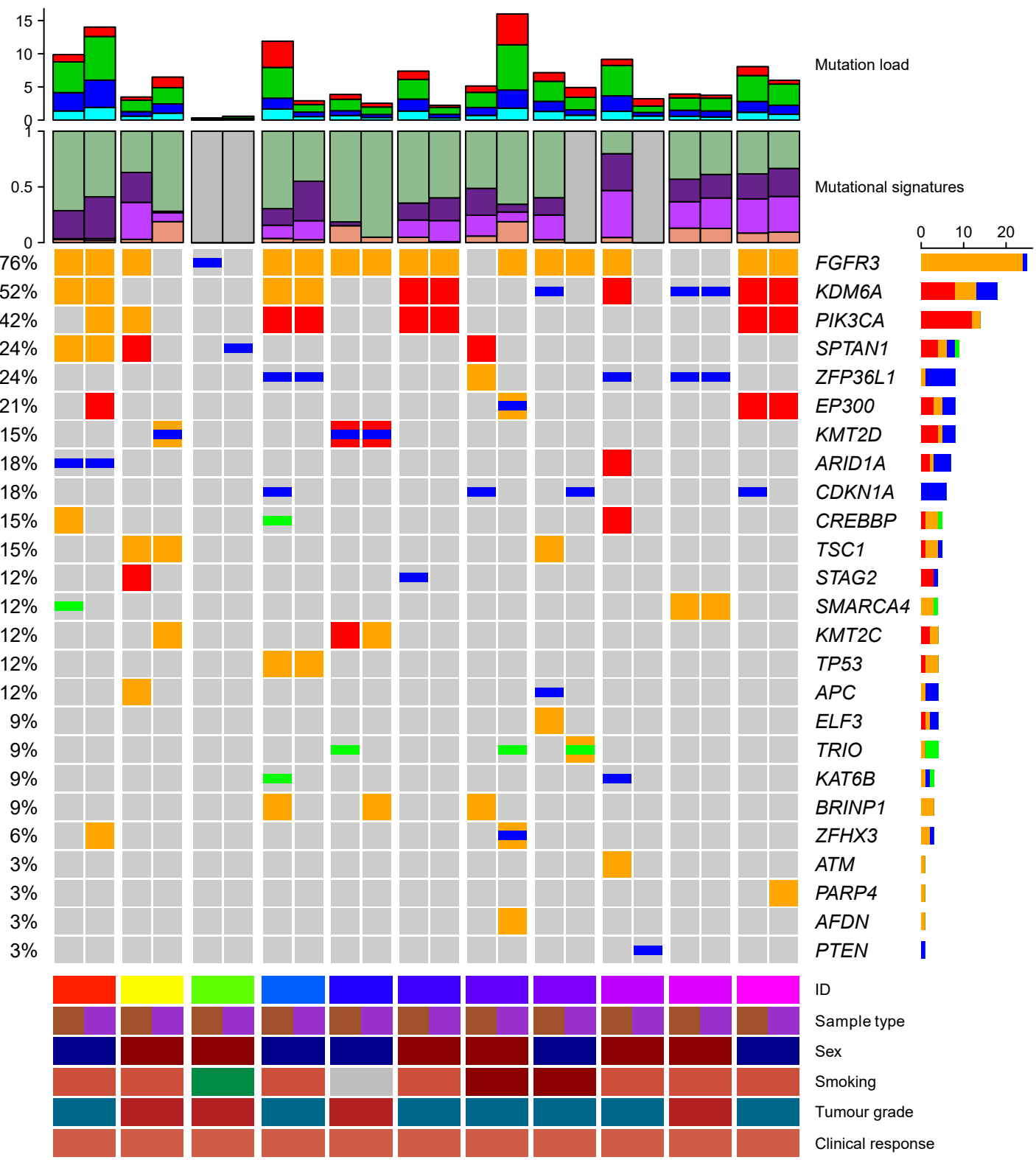

Supplement: Supplementary Fig. 1 — Clinical characteristics and genomic alterations from 11 non-responding patients with paired chemo-naïve and post-chemo samples. A. Oncoplot showing the most frequently mutated genes, known to be bladder cancer-associated in 11 paired chemo-naïve and post-chemo tumour samples. The top panels are annotated by mutation load, which is impact-stratified according to SnpEff and mutational signatures. The bottom panel is annotated by clinical and histopathological characteristics. [file mmc1.pdf]
